# Effect of the association of coagulation/flocculation, hydrodynamic cavitation, ozonation and activated carbon in landfill leachate treatment system

**DOI:** 10.1038/s41598-023-36662-8

**Published:** 2023-06-12

**Authors:** Janaina de Melo Franco Domingos, Thiago de Alencar Neves, Djalma Lucas de Sousa Maia, Rebeca Carvalho Siqueira, Marcus Vinícius Araújo Marques, Oswaldo Luiz Alves, José Roberto Guimarães, Marcelo Antunes Nolasco, André Henrique Rosa

**Affiliations:** 1 School of Arts, Science and Humanities, University of São Paulo (Universidade de São Paulo), São Paulo, Brazil; 2 Department of Sanitary and Environmental Engineering, Federal University of Minas Gerais (Universidade Federal de Minas Gerais), Belo Horizonte, Minas Gerais Brazil; 3 Institute of Science and Technology, Sao Paulo State University (Universidade Estadual de São Paulo), São Paulo, Brazil; 4 Faculty of Civil Engineering, State University of Campinas (Universidade Estadual de Campinas), Campinas, SP Brazil; 5 Technological and Agricultural Center of the State of Bahia (Cetab), Salvador, Bahia Brazil

**Keywords:** Environmental chemistry, Pollution remediation, Engineering

## Abstract

Mature landfill wastewater is a complex effluent due to its low biodegradability and high organic matter content. Currently, mature leachate is treated on-site or transported to wastewater treatment plants (WWTPs). Many WWTPs do not have the capacity to receive mature leachate due to its high organic load leading to an increase in the cost of transportation to treatment plants more adapted to this type of wastewater and the possibility of environmental impacts. Many techniques are used in the treatment of mature leachates, such as coagulation/flocculation, biological reactors, membranes, and advanced oxidative processes. However, the isolated application of these techniques does not achieve efficiency to meet environmental standards. In this regard, this work developed a compact system that combines coagulation and flocculation (1st Stage), hydrodynamic cavitation and ozonation (2nd Stage), and activated carbon polishing (3rd Stage) for the treatment of mature landfill leachate. The synergetic combination of physicochemical and advanced oxidative processes showed a chemical oxygen demand (COD) removal efficiency of over 90% in less than three hours of treatment using the bioflocculant PGα21Ca. Also, the almost absolute removal of apparent color and turbidity was achieved. The remaining CODs of the treated mature leachate were lower when compared to typical domestic sewage of large capitals (COD ~ 600 mg L^−1^), which allows the interconnection of the sanitary landfill to the urban sewage collection network after treatment in this proposed system. The results obtained with the compact system can help in the design of landfill leachate treatment plants, as well as in the treatment of urban and industrial effluents which contains different compounds of emerging concern and persistence in the environment.

## Introduction

Solid waste management in Brazil is a recurring problem, due to inefficient collection and treatment that does not cover the entire population of the country. According to the National Sanitation Information System of Brazil (SNIS), in 2020, around 66.6 million tons of waste were collected, which represents 4.7 million tons more when compared to 2017, and 18% more than in 2010. This scenario reflects a worrying and urgent situation which is the importance of proper management of solid waste. These amounts of waste generated can overload the environmental support capacity^1^.

Among the existing methods for the final disposal of solid waste, sanitary landfills are still frequently used due to their economic advantages and technical complexity. When operated to receive urban solid waste, landfills can receive significant loads of organic matter, an average of 52% of the total, which begins to undergo multiple decomposition reactions, influenced by the infiltration of rainwater and by biochemical processes in the layers of the landfills, forming leachate that percolates through the system. However, within this production flow, several factors can directly and significantly influence the generation and characteristics of the leachate, such as: water content of the residues, precipitation, evaporation, chemical composition of organic and inorganic substances, temperature, pH, among others^1,2^.

According to Mishra et al. (2016), the incorrect handling of the leachate material is one of the biggest contributors to the contamination of soil and surface and groundwater, which reveals the great need for treatment of this material^3^. Inserted in this scenario, the great challenge in the leachate treatment process is to reduce the high concentration of recalcitrant organic compounds, which are difficult to degrade biologically^4^. The presence of these compounds is revealed from the high levels of chemical oxygen demand (COD) and low ratio of biochemical oxygen demand (BOD) to dissolved organic matter (DOM)^1,3,5^.

The physicochemical characteristics and the generated amounts of landfill leachate change with age, where production occurs due to changes between the solid–liquid-gas phases inside the landfill over the years, resulting in a leachate stabilized (BOD/COD < 0.1), with a high load of recalcitrant carbon and low variation in its chemical composition^6^. Leachate is classified according to age, as follows: young landfill leachate (< 5 years), intermediate landfill leachate (between 5 and 10 years), and mature/stabilized landfill leachate (> 10 years)^4,7^.

The degree of stabilization of the landfill leachate is usually evaluated through parameters such as: pH, BOD, COD, TOC, soluble and insoluble fractions of organic matter (such as biodegradable materials and humic/fulvic substances) and characteristics of the biogas composition produced (CO_2_, CH_4_), where the microbial metabolism is the main responsible for reaching the degradation potential of landfilled waste^8,9^.

Aiming to meet environmental regulation standards and minimization of environmental impacts generated, several technologies have been implemented over the years to remove recalcitrant organic residues present in landfill leachate. Among the examples that presented greater treatment efficiencies are coagulation/flocculation, chemical oxidation, adsorption by activated carbon, advanced oxidative processes and processes that involve membrane filtration^4,10^. However, the isolated application of these techniques has proved to be unsatisfactory for the efficient treatment of landfill leachate, requiring a synergistic combination of these processes^11–13^.

Long et al. (2017) in their studies used ferric chloride (FeCl_3_) as a coagulant and demonstrated a variation in COD removal between 82 and 85%^14^. Silva et al. (2017) through a combined treatment consisting of three stages of biological pre-oxidation of aerobic activated sludge (coagulation/sedimentation and photo-oxidation, through a photo-Fenton), reached BOD concentrations below 150 mg L^−1^ ^15^. Gautam et al. (2019) achieved in their studies the removal of 85% in COD when combining ozone with hydrogen peroxide (H_2_O_2_) and persulfate^10^. Other studies using the combination of physicochemical, biological, and oxidative processes for the treatment of landfill leachate are observed in the literature, where the less expressive results are directly related to the treatment of stabilized landfill leachates^11,13^.

Due to the environmental pressure to comply with extremely restrictive standards, aiming to reach an effluent quality compatible for reuse, launching in water courses or in conventional sewage collection systems, a combination of techniques with synergy to generate a high efficiency is hugely necessary. With the evolution of the studies, Advanced Oxidative Processes (AOPs) and Hydrodynamic cavitation emerge as promising strategies to solve the environmental and human health problems of effluents with high treatment complexity^16–18^.

Advanced Oxidative Processes, such as ozonation, have advanced in research using them for leachate treatment, but according to published studies, their exclusive use is not capable of achieving satisfactory results in pollutant removal and treatability^19,20^. One way to enhance the efficiency of treatments is to use them together with other techniques, as examples of this, Feng et al. (2019) used ozonation together with activated carbon and achieved humic acid removal efficiency of 97%^21^.

Hydrodynamic cavitation proved to be a promising technique, used in many fields, such as disinfection, cell rupture, sludge treatment, degradation of organic compounds, however, wastewater treatment is a recent practice and that, together with other technologies, has proven to be promising in increasing efficiencies and reducing treatment costs^22–24^. Research shows that the application of hydrodynamic cavitation in the treatment of landfill leachate in isolation, despite the increase in COD removal efficiency, did not achieve good results, requiring a combination of techniques^18^.

Thus, studies show that the performance results with the combination of ozonation and hydrodynamic cavitation are very promising, however, the lack of studies that can prove their efficiency in the treatment of mature landfill leachate is necessary^25^. The joint use of these technologies mentioned above and the gain when combined with coagulation/flocculation are innovative and necessary.

The poly-y-glutamic acid (PGA) found for commercialization as PGα21Ca, is a bioflocculant that, due to its characteristics of being biodegradable and not causing toxicity to human beings and the environment, has been used in several sectors, among them the treatment of water and wastewater^26–28^. Thus, as an alternative to non-biodegradable coagulants, such as iron and aluminum salts, PGA can be used in the treatment of landfill leachate, which was not found in the literature.

In this context, the present work has as its focus the realization of a systematic, robust and detailed study of the synergistic effect obtained from the combination of techniques in the treatment of stabilized landfill leachate, aimed at reducing treatment time and increasing efficiency. Additionally, this work shows the results of the combination of these technologies, coagulation/flocculation (test of a bioflocculant—PGA), hydrodynamic cavitation, ozonation and the application of activated carbon in the treatment of mature landfill leachate.

## Material and methods

The leachate used in this experiment was collected at the sanitary landfill Delta A, at the geographical coordinates 22° 54′ 47.53″ S and 47° 8′ 35.55″ W, in the municipality of Sorocaba, São Paulo, Brazil, where this landfill no longer receives waste solids, so the leachate generated can be considered stabilized. The experiment was carried out in the laboratories of the State University of Campinas (UNICAMP), São Paulo, Brazil.

To meet the objectives of this work, the characterization of landfill leachate was carried out between the years 2018 and 2021 (Table 1), analyzing the pH, color, turbidity, electrical conductivity (EC), and chemical oxygen demand (COD), carried out according to what was proposed by APHA^29^. The stages proposed for this leachate treatment system were: 1st—Coagulation/Flocculation; 2nd—Hydrodynamic Cavitation plus Ozonation; and 3rd—Activated Carbon, where the efficiencies of the steps were tested according to the color, turbidity and COD analyses, carried out in accordance with APHA^29^.

**Table 1:** Physical–chemical characteristics of raw landfill leachate over the years 2018–2021.

Parameters	Unit	Average	Standard deviation (SD)	Coefficient of variation (CV-%)
pH	–	8.1	0.2	2.2
Color	mg Pt–Co L^−1^	3674.6	861.6	23.4
Turbidity	NTU	10.6	4.9	46.1
COD	mg L^−1^	2167.0	177.1	8.2
EC	mS cm^−1^	12.5	2.0	15.7

n = 13. COD, Chemical oxygen demand; EC, Electrical conductivity.

To meet the 1st Stage of this experiment, four types of coagulants were tested, PGα21Ca, FeCl_3_, AlCl_3_ e Al_2_(SO_4_)_3_, these being considered as treatments of this experiment, the applied dose was 1 g L^−1^, varying the pH between 4 and 8, maintaining fast and slow mixing conditions in a Jar Test system. The best condition results were used for application in the next step.

**Figure 1:**
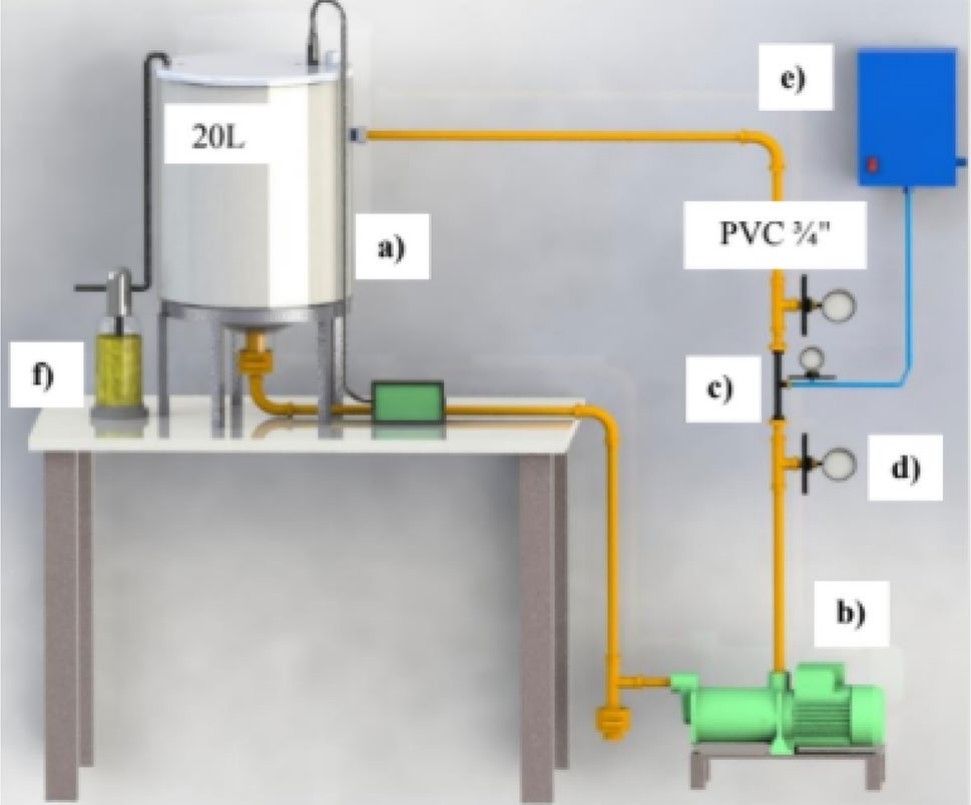
Treatment System 2nd Stage: (**A**)—20 L storage tank; (**B**)—Recirculation Pump (0.12 HP/3420 rpm); (**C**)—Venturi plate with 10 mm diameter concentric orifice; (**D**)—Manometer with operating pressure of 0.59 bar or 0.61 kgf cm^−2^; (**E**)—Ozone generator fed with atmospheric air. (**F**)—KI (Potassium Iodide) solution for off-Gas measurement^30^.

In the 2nd Stage, a system was assembled containing a 20 L reactor coupled to hydrodynamic cavitation, containing a recirculation pump coupled to a Venturi plate with a concentric orifice (10 mm) for extrusion, the system also had an ozonizer, whose application geometry was adapted and patented according to the process “BR 10 2021 025779 2”, this methodology used was based on and adapted from Wu et al. to Bis et al.^7,30^. Ozone (O_3_) generation used the off-gas measurement system in potassium iodide (KI) solution analyzed according to the Iodometric Method 2350 E^31^. Figure 1 shows the illustration of the 2nd Stage system.

The 2nd Stage was operated in a closed circuit (366 mg O_3_ L^−1^), therefore, before testing the complete system (all stages), tests were carried out to define the operating time without application of the coagulant, being tested 1, 2, 3, 4, 5 and 6 h, where after each test the effluent was passed through a system that simulated a conventional decanter. The best results were applied to the complete system.

In the 3rd Stage, 1 g L^−1^ of powdered activated carbon was applied in a rapid mixing system to maintain the contact time, after which it was passed through a system that simulated a conventional decanter. This step was the last of this experiment, where the results were used to define the total efficiency of the system.

Statistical analyses consisted of comparative tests of central tendencies, using the analysis of variance (ANOVA) followed by the Tukey test, in which the level of significance was set at 5%, comparing the total efficiencies of the system (all steps).

## Results and discussion

### Mature leachate physicochemical characterization

The leachate produced in mature landfills is characterized by the considerable biodegradability difference from young and medium-age landfill leachates due to the stabilization process of the urban solid waste. The mature leachate generally has a stable physicochemical composition, for instance high concentration of refractory organics compounds (e.g., humic and fulvic substances). Due to these characteristics, the first stage of this work was to characterize the landfill to better understand it and define the appropriate treatment methods to be applied. The sampling campaign comprised the wet and dry season throughout 3 years of study. Table 1 presents the physicochemical characteristics of the raw leachate from the landfill studied.

The pH is the parameter that influences the coagulation/flocculation conditions and varied between 7.9 and 8.5 in untreated mature landfill leachate, similar conditions were found in other studies^4,7,32–37^. This low variation (CV—2.2%) and the tendency towards basicity of this effluent is common due to the natural stabilization through which the medium undergoes over time, because of the decrease in the concentration of partially ionized free volatile fatty acids, which are consumed by methane-producing bacteria^31^. Thus, as with the pH values, the results obtained in the other parameters were very close to studies with similar stabilization conditions, such as those carried out in Florida (USA) and also in Croatia^35,38^.

**Figure 2:**
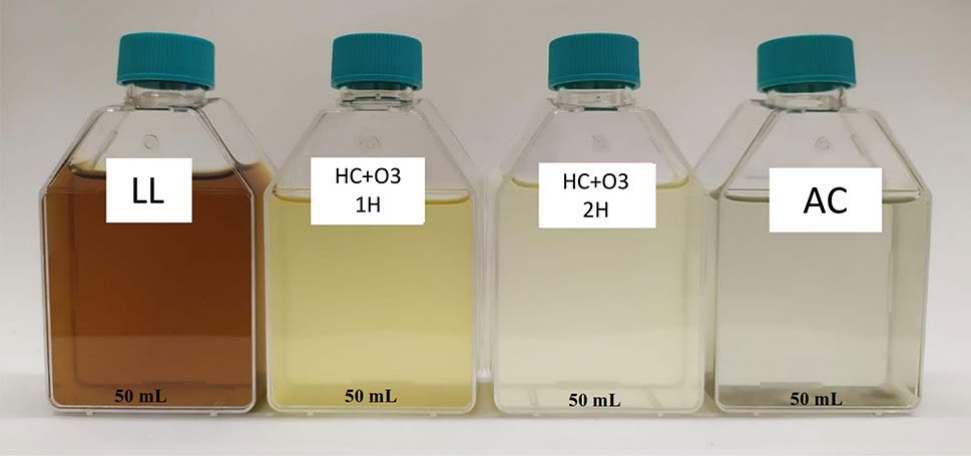
Color variation of landfill leachate after application of the various processes when using the coagulant Al_2_(SO_4_)_3_. LL, Landfill leachate; HC + O_3_ 1H, Hydrodynamic cavitation plus Ozonation after one hour test; HC + O_3_ 2H, Hydrodynamic cavitation plus Ozonation after two hours test; AC, Activated carbon.

The color parameter (Table 1) expressed as apparent color (mg Pt–Co L^−1^) presented high concentrations values when compared with different mature landfills in the literature. As mentioned, with age the landfill changes its composition and becomes dominated by refractory compounds. The dark brownish color (Fig. 2) is due to the presence of high concentration of DOM compounds which are correlated to the relative abundance of aromatic substances and chromogenic functional groups. The presence of high organic concentrations can become a serious environmental problem manly because color hinders the growth of aquatic life by decreasing the sunlight penetration, subsequently disturbing photosynthetic activity. The values of apparent color and turbidity are important to the coagulation and flocculation process due to the colloidal concentration fraction in the mature leachate^39–42^.

The turbidity parameter (Table 1) showed the highest coefficient of variation and low values when compared with other types of wastewaters (e.g., domestic sewage). Low colloidal or suspend particle concentrations can affect the coagulant dosage and charge neutralization in the landfill treatment process. Also, the low values and variation of turbidity can be associated to the high concentrations of apparent color which can affects the readings of the nephelometric method.

Studies show that the COD value of landfill leachate is usually below 10,000 mg L^−1^, where the lower this value, the older is the leachate^4^, therefore, the leachate studied in this work is in an advanced stage of stabilization. After 10 years, a landfill will be composed with less biodegradable organic matter, influencing the COD of the leachate for values below 4000 mg L^−1^. When it reaches this stage, the organic matter is composed of complex macromolecular organic compounds that present significant resistance to biodegradation and is characterized as a refractory organic wastewater. Therefore, the leachate will present high pH values, ammoniacal nitrogen concentration, low CH_4_ production and BOD_5_/COD ratio less than 0.1^37,43–45^. In the present study, a BOD_5_/COD ratio of less than 0.06 was observed from the database provided by the company responsible for monitoring the landfill under study.

Accordingly, to Chen et al. 2020 and Liu 2022 the DOM presented in the mature leachate has unbalanced carbon-to-nitrogen ratio, and high molecular weight (highly aromatic benzene ring compounds) and high degree of unsaturation compounds which can cause great difficulty to be treated. The data observed by Chen et al. 2020, Liu et al. 2022 and Gu et al. 2022 shows that mature leachate DOM classes of compounds possess the relative proportions of phenolic compounds > aliphatic compounds > polyphenols > polycyclic aromatics based on van Krevelen diagrams. In addition, Liu et al. 2022 investigated and compared the molecular weight distribution of DOM in mature and young leachate. The authors conclude that the molecular weight distribution of DOM in mature leachate was wider and contained more DOM with molecular weights greater than 400 m/z. Whereas, for young leachate the distribution range of DOM was smaller and the density center of the molecular weight distribution of DOM was significantly lower than that of the mature leachate^39,40,46^. Kulikowska & Klimiuk (2008) report that there is significant variation in the quality of the leachate produced in different sanitary landfills around the world, even among those considered mature for chemical composition, in particular, for organic compounds and nitrogen^47^.

Therefore, the characteristics of the leachate used in this work (Table 1) show that the traditional treatment methods reported in the literature are not efficient to treat this type of effluent, justifying the need for a new treatment process based on the combination of advanced techniques to obtain a satisfactory result.

### Operational parameters of coagulation and hydrodynamic cavitation plus ozonation

Based on the characterization of the mature leachate, physicochemical techniques and advanced oxidative processes were chosen to evaluate the removal efficiency of the high concentrations of COD and apparent color. Initially, coagulation/flocculation processes were tested using traditional chemical coagulants (AlCl_3_, Al_2_SO_4_, FeCl_3_) and compared with a bioflocculant (Poly-Glu—PGα21Ca) proposed for the treatment of leachate. Similarly, hydrodynamic cavitation associated with ozonation were tested and compared with the coagulation/flocculation in terms of removal efficiency of COD and apparent color.

There are several studies regarding pH in optimizing coagulation in the production of drinking water and in the treatment of effluents, combining the ideal pH and system conditions with the efficiency of pollutant removal^48^. More recently, the application of coagulation/flocculation processes has proven to be a successful method for the removal of contaminants that can be adsorbed by colloids such as toxic organic matter, viruses and metals. Coagulation/flocculation is necessary to reduce the colloidal fraction, the total suspended solids and color in order to improve the efficiency of subsequent treatment process. The results found in this study show that for FeCl_3_, AlCl_3_ e Al_2_(SO_4_)_3_ the ideal pH value is close to 5.0 and for PGα21Ca the ideal pH would be the range of 4.0. Trivalent salts of aluminum and iron, produce many species by hydrolysis producing positively charged metallic hydroxyoxide complexes (e.g., Al_6_(OH)_15_^3+^ and Fe_3_(OH)_4_^5+^). These hydrolysis species act on charge neutralization of negatively charged colloids which permits the van der Waals force of attraction to induce colloidal particle aggregation to form flocs.

When FeCl_3_ was used, the COD reduction reached 56.8% and the color 39.5% efficiency, with AlCl_3_ the COD removal efficiency was 62.4% and the color 74.6%, with the coagulant PGα21Ca the removal was 52.5% for COD and the color 76.3%, and when Al_2_(SO_4_)_3_ was used the COD removal was 52.0% and the color 64.4% under the indicated pH conditions. Different factors have impact on the coagulation/floculation efficency, such as the chemical compositon of coagulant, the coagulation pH, and dosage. To obttain high effiency of treatment is mandatory to evaluate the combination of these factors. In a study carried out in Malaysia using ferric sulfate in a coagulation/flocculation test of leachate from a landfill, at a dosage of 5500 mg L^−1^ at pH 6, it was possible to achieve a removal of 47% of color in the study^38^. This variation indicates that with the test of new coagulants in different dosages it is possible to improve the percentages of color removal and consequently the COD at this stage^38^.

To date, very few studies were reported on the application of PGα21Ca in the coagulation/flocculation of landfill leachate, in a study in the treatment of wastewater from the production of potato starch, it was found that the pH equal to 4.0 presented the best results, like this study^26^. In a study carried out with vinasse, even working with temperatures above natural, they show that the best coagulation conditions with PGα21Ca are with a pH below 4.0^27^. In terms of COD removal, the PGα21Ca has shown higher efficiency than FeCl_3_, AlCl_3_ e Al_2_(SO_4_)_3_ salts. This can be explained because PGα21Ca has in addition to γ-PGA in its composition, calcium and aluminum sulfate/carbonates acting as auxiliary coagulants.

The Table 2 presents the results of the hydrodynamic cavitation tests together with ozonation, without the application of coagulants to define the best operating time for the system. The table shows that the increase in removal efficiency in percentage occurred more significantly from the 1st to 2nd hour of the test, with an increase of 15.2% for COD, 27.9% for turbidity and 11.5% for color. The reduction of COD can be explained because hydrodynamic cavitation is based in the formation, growth, and collapse of vapor cavities induced by a restrictive flow device such as a pump, jet nozzle, propeller, or orifice plate^49–51^. As cavities collapse, bubble formation can reach elevated temperatures and pressures. This process, called hot spots, it is capable to produce turbulence, highly reactive free radicals, and high-speed jets within the fluid. When associated to ozonation the process contributes for rapid absorption of ozone molecules in the system. The combined process of cavitation with ozone produces ·OH radicals therefore can significantly improve the degradation efficiency of the process^18,22,52^.

**Table 2:** Average results of reactor operation tests with hydrodynamic cavitation and ozonation as a function of system operation time without coagulant application.

Parameters	LL	1 h	2 h	3 h	4 h	5 h	6 h
Color (mg Pt–Co L^−1^)	3697	913.3	489.7	375.7	225.7	185.3	188.7
Efficiency (%)		75.3	86.8	89.8	93.8	95.0	94.9
Turbidity (NTU)	11.1	9.0	5.9	4.7	4.0	3.3	3.3
Efficiency (%)		18.9	46.8	57.7	61.7	69.1	69.4
COD (mg L^−1^)	2017	1676.9	1369.8	1261.5	1302.2	1208.2	1288.0
Efficiency (%)		16.9	32.1	37.5	35.4	40.1	36.1

Triplicate analysis (n = 3). COD, Chemical oxygen demand. LL, Landfill leachate.

The increments from the 2nd to 3rd hour of system operation in efficiency were smaller compared to the increment from the 1st to 2nd hour, being 5.4% for COD, 10.8% for turbidity and 3.1%, being similar in the other times tested (Table 2). Similar observations were reported by Huo et al. 2008, Gutiérrez-Mosquera et al. 2022 and Wang et al. 2021 which can be associated with the decreased fluid flow causing the cavitation device to be filled with water, leading to higher static pressure and early collapse of cavities^18,22,52^. Also, Huo et al. 2008 observed that longer operational times can drive aromatic polycondesantion which increases the DOM humidification causing an increase in the COD values in the system^52^. The study of Wang et al. 2021 obtained data showing that venturi-type devices can ensure a higher throat velocity for a given pressure drop, which can generate a lower cavitation number per operational time^22^. After the second hour of treatment the efficiencies removal of the parameters reached a state of greater system stability where trends in organic matter removal were not observed. With these results it was defined that the operating time of the 2nd Stage, hydrodynamic cavitation plus ozonation was two hours in the complete tests (all stages).

### Combined treatment of mature leachate

After analyzing the processes individually, a study was carried out to evaluate the synergistic effect of physicochemical and advanced oxidative processes. From the results shown in Table 3, it can be observed a high total removal efficiency in the COD (greater than 80%) for all types of applied coagulants, it also shows the efficiency part of the 2nd stage between the 1st and 2nd hour of application of hydrodynamic cavitation plus ozonation and polishing with activated carbon.

**Table 3:** COD, Color and Turbidity results for each stage of the proposed landfill leachate treatment system, the coagulation/flocculation (CF), hydrodynamic cavitation (HC) plus ozonation (O_3_) and activated carbon (AC) application.

Parameters	PGα21Ca–pH 4			AlCl_3_–pH 5			FeCl_3_–pH 5			Al_2_(SO_4_)_3_–pH 5		
	AV	SD	EF (%)	AV	SD	EF (%)	AV	SD	EF (%)	AV	SD	EF (%)
COD (mg L^−1^)												
LL	2425			2425			2425			2209		
CF	1466	118	39.5	1595	266	34.2	1731	253	28.6	1444	486	34.6
HC + O_3_ 1H	702	86.8	31.5	722	84.0	36.0	770	179	39.6	798	181	29.2
HC + O_3_ 2H	662	72.0	1.6	582	81.3	5.8	642	175	5.3	543	191	11.5
AC	183	10.1	19.8	347	18.3	9.7	444	68.4	8.2	369	22.5	7.9
Total			92.5B			85.7A			81.7A			83.3A
Color (mg Pt–Co L^−1^)												
LL	3760			3840			3840			4080		
CF	1462	171	59.1	1537	484	60.0	2857	25	25.6	2903	535	28.8
HC + O_3_ 1H	425	70.9	33.2	203	50.3	34.7	1053	491	47.0	637	211	55.6
HC + O_3_ 2H	202	30.6	3.3	143	15.3	1.6	710	451	8.9	347	86.2	7.1
AC	170	21.2	2.1	102	24.3	1.1	383	145	8.5	103	70.9	6.0
Total			97.7B			97.3B			90.0A			97.5B
Turbidity (NTU)												
LL	11.2			11.2			11.2			10.4		
CF	20.0	1.9	− 78.3	10.6	2.0	5.8	14.0	3.7	− 25.0	20.3	3.1	− 95.5
HC + O_3_ 1H	1.7	0.3	162.8	1.6	0.4	79.7	9.4	1.2	41.5	13.0	4.7	70.6
HC + O_3_ 2H	1.3	0.04	4.0	1.4	0.4	2.1	11.4	2.2	− 18.4	5.7	0.2	70.2
AC	3.9	1.2	− 22.9	4.1	1.6	− 24.3	15.1	3.0	− 32.6	2.5	1.7	30.3
Total			65.6B			63.2B			− 34.5A			75.6B

Treatments with the same letter have no significant difference according to the Tukey test (p < 0.05, n = 12). LL, Landfill leachate; CF, Coagulation/flocculation; HC + O_3_ 1H, Hydrodynamic cavitation plus Ozonation after one hour test; HC + O_3_ 2H, Hydrodynamic cavitation plus Ozonation after two hours test; AV, Average; SD, Standard deviation; EF, Efficiency; COD, Chemical oxygen demand.

The stablished condition for the coagulation/flocculation process contributed to the removal of organic substances and chromaticity which can be observed in the reduction of COD and apparent color. The differences in efficiency removal of color and COD between the coagulants used can be explained by the interaction of DOM with different types of flocculants. As stated by Wang et al. 2022 aluminum salt flocculants preferentially remove unsaturated larger compounds and iron salt can remove smaller saturated compounds. This could explain the lower efficiency of FeCl_3_ in the studied experimental conditions.

The combination of coagulation/flocculation with hydrodynamic cavitation/ozonation was effective to reduce refractory organic matter concentration and color in mature landfill leachate. The color reduction can be associated with the destruction of chromophores and auxochrome groups, and the organic-containing benzene ring structures as appointed by Chen et al.^39^. Also, since ozone is very effective in the oxidation of aromatic compounds susceptible to electrophilic attack, the cavitation process can enhance the interaction of DOM with ·OH radicals formed in the systems, thus significantly improving the degradation efficiency.

Among the coagulants studied, PGα21Ca was the one that presented the highest total efficiency of COD removal from the crude leachate, which initially had an average concentration of 2425 mg L^−1^ and at the end of 183 mg L^−1^ of COD, that is, treatment efficiency of 92.5%, being significantly different from other coagulants. The second coagulant that showed the best performance in reducing COD was AlCl_3_ with 85.7%, followed by Al_2_(SO_4_)_3_ with 83.3% and finally FeCl_3_ showing a reduction of 81.7%, however this did not show significant difference between each other (Table 3).

There are records in the literature of the use of bioflocculants, such as *Ocimum basilicum*, extracted from the basil seed, together with ozone for the treatment of landfill leachate at doses of 0.2 g O_3_ L^−1^ h^−1^ during 30 min. The technique carried out in the province of Guilan (Iran), integrated coagulation/flocculation with ozonation and obtained an efficiency of 87% and 92% of COD and color, respectively^53^.

Through the data in Table 3, it can be observed that the best results obtained for reducing color and turbidity were achieved from the use of PGα21Ca, a compound recently used in water treatment plants for supply, with a significant difference when compared to with FeCl_3_. The PGα21Ca is a product developed by Nippon Poly-Glu Co and obtained from Bacillus licheniformis and Bacillus subtilis through fermentative processes. It is a natural bioflocculant mixture containing γ-PGA with cross-link product of γ-PGA with the addition of calcium sulfate, calcium carbonate, sodium bicarbonate and aluminum sulfate. The γ-PGA is anionic polymer which is ideal for colloid removals and DOM compounds. With the addition of alkalinity species and coagulants, such as aluminum, the PGα21Ca is a versatile flocculant and can be applied in wide range of pH. The mechanism of action of PGα21Ca can be explained firstly by the effect of the agglomerating of particles and suspended solids carried out by the presence of aluminum salts and then by the increase in interaction and colloidal nucleation stimulated by γ-PGA. This process allows the formation of denser aggregates and heavy flakes^28,54,55^.

When FeCl_3_ was used, the results were not satisfactory, and in the end the effluent had a cloudy, yellowish appearance, which influenced its turbidity to remain present even after the adsorption of activated carbon. The turbidity efficiency result of the system that used FeCl_3_ was negative, going from 11.2 to 15.1 NTU, an increase of 34.5% (Table 3).

As shown, the color of the effluent after using FeCl_3_ was not satisfactory, but when COD removal was observed, the scenario was different. According to Oloibiri et al. (2015) the time required for the treatment of leachate by coagulation–flocculation, with the coagulant in question, associated with the adsorption of activated carbon, is 258 min to achieve a reduction of 53% of COD^33^. As for the combination of ozonation and adsorption by activated carbon, it took 240 min to reach a removal of 77%. In the case of the present study, it took 200 min to reach a COD removal of 81.7%, that is, both the time and the efficiency of organic matter removal were superior to the study described above.

As mentioned above, the use of the AlCl_3_ coagulant provided a COD removal of 85.7%, a high result (greater than 80%) in a leachate treatment with high levels of contaminants. A study carried out with treatment by coagulation and flocculation associated with ozonation, with stabilized leachate and already coming from secondary treatments (anaerobic and facultative pond), the final removal of COD was close to 72%, a value lower than that achieved by the present study^56^. In comparison, another study in the treatment of landfill leachate, used coagulation–flocculation, with polymeric ferric sulfate (250 mg L^−1^), combined with ozonation, at the end of the process an efficiency of 88.3% of COD was obtained^57^.

Rivas et al. (2003) carried out a study similar to the present work, the leachate used had already been submitted to the ozonation process, then it went through the adsorption process with activated carbon (30 g L^−1^) during the period of 120 min, under these conditions a COD removal efficiency of 90% was reached. It should be emphasized that in the present study it was possible to reduce COD values in an efficiency range between 81.7 to 87.2% using only 1 g L^−1^ of activated charcoal and 200 min of treatment^58^.

Figure 2 shows an image of the raw leachate and its evolution as the treatment progresses, in which Al_2_(SO_4_)_3_ was used as a coagulant and showed high removal efficiency in terms of color. The initial dark brown coloration can be attributed to the presence of humic substances, which are gradually lost due to the dosages and time of exposure to ozone, changing to a light-yellow color until it becomes almost colorless after the application of activated carbon. This same evolution was observed and reported in the study carried out by Ntampou et al.^56^.

The work by Gottshalk et al. (2020), Rivas et al. (2003) and Silva et al. (2004) described that the removal of color may be related to the direct attack of ozone on the double bonds of carbon atoms located in the chromophore groups of the compounds comprising the leachate, in addition to the attack itself on the aliphatic bonds, ketones and aldehydes^58–60^. In summary, all coagulants showed little differentiation regarding the removal efficiency of the parameters monitored in the treatability tests, with the exception of ferric chloride, which showed lower removal levels. Currently, there has been a concern about the management of coagulants used in wastewater treatment in general (sludge volume), however, studies are more comprehensive for sludge generated in supply water treatment, which is known to have few toxic pollutants. In any case, the ways of managing the coagulation sludge can be the use as a substrate in constructing wetlands, building materials (ceramic, brick, and cement chemicals), abacation of Geopolymers (GPs) and removal of pollutants^61^. Table 4 shows the efficiencies of mature landfill leachate treatments using processes that involve the synergy of physicochemical treatments with advanced oxidative processes.

**Table 4:** Performance comparison of landfill leachate treatment for technologies correlated to Flocculation/Coagulation and Advanced Oxidative Process.

Effluent/country	Type	COD removal efficiency (%)	Initial COD (mg L^−1^)	BOD_5_/COD	References
Stabilized LL (Brazil)	CF + HC + O_3_ + AD	92.5	2167	0.06	Present study
Stabilized LL (China)	CF + AD	86	2817	0.05	Li et al.^62^
Stabilized LL (Malaysia)	CF + UV	91.5	5123	0.07	Ishak et al.^12^
Stabilized LL (China)	MT + CF + O_3_	88.3	3855	0.003	Chen et al.^57^
Stabilized LL (Malaysia)	O_3_ + CF	88	4899	0.25	Zakaria et al.^11^
Stabilized LL (Portugal)	CF + FT	89	5700	0.07	Amor et al.^63^
Stabilized LL (Colombia)	HC + H_2_O_2_	38.2	34,405	0.08	Gutiérrez-Mosquera et al.^18^
Stabilized LL (Morocco)	CF + H_2_O_2_ + PS + US + UV	89	3669	0.09	Bellouk et al.^64^

NI, Not Included; CF, Coagulation/Flocculation; HC, Hydrodynamic Cavitation; O_3_, Ozonation; AD, Adsorption; H_2_O_2_, Hydrogen peroxide; US. Ultrasound; PS, Potassium Persulfate; MT, Membrane Treatment; UV, Ultraviolet; FT, Fenton.

It can be seen that the combination of coagulation/flocculation with advanced oxaditive processes is efficient for COD removal of mature landfill leachate with different BOD_5_/COD levels. Removal efficiencies greater than 86% can be observed, however treatment time, energy costs and infrastructure are factors that need to be considered when applying these processes on full scale.

## Conclusion

The combination and association of techniques of coagulation/flocculation, hydrodynamic cavitation, ozonation and activated carbon were efficient for the treatment of mature landfill leachate with the use of different coagulants, with emphasis on PGα21Ca as greater efficiencies.

The operational parameters defined in this work show that in coagulation/flocculation for FeCl_3_, AlCl_3_ and Al_2_(SO_4_)_3_ the ideal pH value is close to 5.0 and for PGα21Ca the ideal pH would be the range of 4.0. In hydrodynamic cavitation/ozonation, the ideal operating time was close to 2 h, with the first hour showing the greatest increase in efficiency.

The coagulants evaluated and used in the coagulation/flocculation stage of the landfill leachate treatment of the proposed system, showed that the efficiency of DOC removal were all greater than 80%, with emphasis on the coagulant PGα21Ca, with removal of 92.5% of COD, followed by AlCl_3_ with 85.7%, Al_2_(SO_4_)_3_ with 83.3% and FeCl_3_ with the lowest performance, with removal of 81.7% of COD, with a significant difference between PGα21Ca and the other coagulants.

The color removal in the proposed system was higher with the application of the PGα21Ca coagulant, when compared with the FeCl_3_, being 97.7% and 90%, respectively. About turbidity, the system with the application of FeCl_3_ coagulant was not satisfactory, being significantly lower than the other treatments.

The results obtained show that the proposed system can significantly reduce the concentration of organic matter in the treated leachate, making it possible to release and dilute it in the urban sewage collection network, considering that in large cities the COD values of 600 mg L^−1^ are normally found.

It is noteworthy that many mature sanitary landfills need to send their leachate for treatment in effluent treatment plants, where several requirements are required to receive this type of waste. In this scenario, the implementation of the proposed system can contribute to solve this problem.

## Data Availability

All data generated or analyzed during this study are included in this published article in the form of figures, tables, and graphs.

## References

[1] Moersidik SS, Annasari L, Nugroho R (2021). Application of cavitation ozonation process on recalcitrant organic matter degradation from stabilized landfill leachate. Int. J. Technol..

[2] Nascimento MCB, Freire EP, de Dantas FAS, Giansante MB (2019). Estado da arte dos aterros de resíduos sólidos urbanos que aproveitam o biogás para geração de energia elétrica e biometano no Brasil. Engenharia Sanitaria e Ambiental.

[3] Mishra H, Karmakar S, Kumar R, Singh J (2017). A framework for assessing uncertainty associated with human health risks from MSW landfill leachate contamination. Risk Anal..

[4] Luo H, Zeng Y, Cheng Y, He D, Pan X (2020). Recent advances in municipal landfill leachate: A review focusing on its characteristics, treatment, and toxicity assessment. Sci. Total Environ..

[5] Ghanbari F, Wu J, Khatebasreh M, Ding D, Lin K-YA (2020). Efficient treatment for landfill leachate through sequential electrocoagulation, electrooxidation and PMS/UV/CuFe2O4 process. Sep. Purif. Technol..

[6] Chen Y-C (2016). Potential for energy recovery and greenhouse gas mitigation from municipal solid waste using a waste-to-material approach. Waste Manag..

[7] Bis M, Montusiewicz A, Ozonek J, Pasieczna-Patkowska S (2015). Application of hydrodynamic cavitation to improve the biodegradability of mature landfill leachate. Ultrason. Sonochem..

[8] Pearse LF, Hettiaratchi JP, Kumar S (2018). Towards developing a representative biochemical methane potential (BMP) assay for landfilled municipal solid waste—A review. Bioresour. Technol..

[9] Zhao Y, Lou Z (2017). Pollution Control and Resource Recovery: Municipal Solid Wastes at Landfill.

[10] Gautam P, Kumar S, Lokhandwala S (2019). Advanced oxidation processes for treatment of leachate from hazardous waste landfill: A critical review. J. Clean. Prod..

[11] Zakaria SNF, Abdul Aziz H, Mohamad M (2022). Comparison performance of coagulation flocculation process and combination with ozonation process of stabilized landfill leachate treatment. Water Environ. Res..

[12] Ishak AR, Hamid FS, Mohamad S, Tay KS (2018). Stabilized landfill leachate treatment by coagulation–flocculation coupled with UV-based sulfate radical oxidation process. Waste Manag..

[13] Babaei S, Sabour MR, Moftakhari Anasori Movahed S (2021). Combined landfill leachate treatment methods: An overview. Environ. Sci. Pollut. Res..

[14] Kumwimba MN, Zhu B, Suanon F, Muyembe DK, Dzakpasu M (2017). Long-term impact of primary domestic sewage on metal/loid accumulation in drainage ditch sediments, plants and water: Implications for phytoremediation and restoration. Sci. Total Environ..

[15] Silva TFCV (2017). An innovative multistage treatment system for sanitary landfill leachate depuration: Studies at pilot-scale. Sci. Total Environ..

[16] Tousizadeh S, Arbabi M, Tondro E, Sedehi M, Arbabi A (2022). Evaluation of chemical oxygen demand and color removal from leachate using coagulation/flocculation combined with advanced oxidation process. Adv. Biomed. Res..

[17] Lin R (2022). Synergistic effects of oxidation, coagulation and adsorption in the integrated fenton-based process for wastewater treatment: A review. J. Environ. Manag..

[18] Gutiérrez-Mosquera LF, Arias-Giraldo S, Zuluaga-Meza A (2022). Landfill leachate treatment using hydrodynamic cavitation: Exploratory evaluation. Heliyon.

[19] Wu C, Chen W, Gu Z, Li Q (2021). A review of the characteristics of Fenton and ozonation systems in landfill leachate treatment. Sci. Total Environ..

[20] Amaral-Silva N, Martins RC, Castro-Silva S, Quinta-Ferreira RM (2016). Ozonation and perozonation on the biodegradability improvement of a landfill leachate. J. Environ. Chem. Eng..

[21] Feng J, Xing B, Chen H (2019). Catalytic ozonation of humic acid in water with modified activated carbon: Enhancement and restoration of the activity of an activated carbon catalyst. J. Environ. Manag..

[22] Wang B, Su H, Zhang B (2021). Hydrodynamic cavitation as a promising route for wastewater treatment—A review. Chem. Eng. J..

[23] Hong F (2022). CFD-assisted modeling of the hydrodynamic cavitation reactors for wastewater treatment—A review. J. Environ. Manag..

[24] Kundu A, Reddy CV, Singh RK, Kalamdhad AS (2023). Critical review with science mapping on the latest pre-treatment technologies of landfill leachate. J. Environ. Manag..

[25] Wang J (2022). The advanced treatment of textile printing and dyeing wastewater by hydrodynamic cavitation and ozone: Degradation, mechanism, and transformation of dissolved organic matter. Environ. Res..

[26] Li M (2020). Treatment of potato starch wastewater by dual natural flocculants of chitosan and poly-glutamic acid. J. Clean. Prod..

[27] Carvajal-Zarrabal O (2012). Treatment of vinasse from tequila production using polyglutamic acid. J. Environ. Manag..

[28] Campos V, Fernandes ARAC, Medeiros TAM, Andrade EL (2016). Physicochemical characterization and evaluation of PGA bioflocculant in coagulation–flocculation and sedimentation processes. J. Environ. Chem. Eng..

[29] APHA—American Public Health Association. in*Standard Methods for the Examination of Water ans Wastewater*. (2017).

[30] Wu Z (2012). Removal of blue-green algae using the hybrid method of hydrodynamic cavitation and ozonation. J. Hazard Mater..

[31] Singh SK, Moody CM, Townsend TG (2014). Ozonation pretreatment for stabilized landfill leachate high-pressure membrane treatment. Desalination.

[32] Chys M, Declerck W, Audenaert WTM, van Hulle SWH (2015). UV/H2 O2, O3 and (photo-) Fenton as treatment prior to granular activated carbon filtration of biologically stabilized landfill leachate. J. Chem. Technol. Biotechnol..

[33] Oloibiri V (2015). A comparative study on the efficiency of ozonation and coagulation–flocculation as pretreatment to activated carbon adsorption of biologically stabilized landfill leachate. Waste Manag..

[34] Dolar D, Košutić K, Strmecky T (2016). Hybrid processes for treatment of landfill leachate: Coagulation/UF/NF-RO and adsorption/UF/NF-RO. Sep. Purif. Technol..

[35] Jung C, Deng Y, Zhao R, Torrens K (2017). Chemical oxidation for mitigation of UV-quenching substances (UVQS) from municipal landfill leachate: Fenton process versus ozonation. Water Res..

[36] Hussein M, Yoneda K, Zaki ZM, Othman N, Amir A (2019). Leachate characterizations and pollution indices of active and closed unlined landfills in Malaysia. Environ. Nanotechnol. Monit. Manag..

[37] Assou M (2016). Landfill leachate treatment by a coagulation–flocculation process: Effect of the introduction order of the reagents. Desalin. Water Treat..

[38] Aziz H (2007). Colour removal from landfill leachate by coagulation and flocculation processes. Bioresour. Technol..

[39] Chen W, Zhuo X, He C, Shi Q, Li Q (2020). Molecular investigation into the transformation of dissolved organic matter in mature landfill leachate during treatment in a combined membrane bioreactor-reverse osmosis process. J Hazard Mater..

[40] Gu Z, Chen W, He C, Li Q (2022). Molecular insights into the transformation of refractory organic matter in landfill leachate nanofiltration concentrates during a flocculation and O3/H2O2 treatment. J. Hazard Mater..

[41] Karimipourfard D, Eslamloueyan R, Mehranbod N (2019). Novel heterogeneous degradation of mature landfill leachate using persulfate and magnetic CuFe2O4/RGO nanocatalyst. Process. Saf. Environ. Prot..

[42] Bashir MJK (2009). Landfill leachate treatment by electrochemical oxidation. Waste Manag..

[43] Aziz SQ, Aziz HA, Yusoff MS, Bashir MJK, Umar M (2010). Leachate characterization in semi-aerobic and anaerobic sanitary landfills: A comparative study. J. Environ. Manag..

[44] Nair A, Sartaj M, Kennedy K, Coelho NM (2014). Enhancing biogas production from anaerobic biodegradation of the organic fraction of municipal solid waste through leachate blending and recirculation. Waste Manag. Res. J. Sustain. Circ. Econ..

[45] Renou S, Givaudan JG, Poulain S, Dirassouyan F, Moulin P (2008). Landfill leachate treatment: Review and opportunity. J. Hazard Mater..

[46] Liu J, Gu Z, Wang X, Li Q (2022). The molecular differences of young and mature landfill leachates: Molecular composition, chemical property, and structural characteristic. Chemosphere.

[47] Kulikowska D, Klimiuk E (2008). The effect of landfill age on municipal leachate composition. Bioresour. Technol..

[48] Naceradska J, Pivokonska L, Pivokonsky M (2019). On the importance of pH value in coagulation. J. Water Supply Res. Technol. AQUA.

[49] Lohrberg H, Stoffel B, Voss B, Glesner M (2000). Impeller integrated measurement of cavitation erosive aggressiveness. IFAC Proc..

[50] Gogate PR, Pandit AB (2001). Hydrodynamic cavitation reactors: A state of the art review. Rev. Chem. Eng..

[51] Balasundaram B, Harrison STL (2006). Study of physical and biological factors involved in the disruption of *E. coli* by hydrodynamic cavitation. Biotechnol Prog.

[52] Huo S (2008). Characteristics of dissolved organic matter (DOM) in leachate with different landfill ages. J. Environ. Sci..

[53] Rasool MA, Tavakoli B, Chaibakhsh N, Pendashteh AR, Mirroshandel AS (2016). Use of a plant-based coagulant in coagulation–ozonation combined treatment of leachate from a waste dumping site. Ecol. Eng..

[54] Campos, V., Domingos, J. M. F., Dos Anjos, D. N. & Lira, V. S. Study of fluvial water treatability using γ-polyglutamic acid based biopolymer coagulant. *An. Acad. Bras. Cienc.* **91**, (2019). 10.1590/0001-3765201920190051 31482995

[55] Pooi CK, Ng HY (2018). Review of low-cost point-of-use water treatment systems for developing communities. NPJ Clean. Water.

[56] Ntampou X, Zouboulis AI, Samaras P (2006). Appropriate combination of physico-chemical methods (coagulation/flocculation and ozonation) for the efficient treatment of landfill leachates. Chemosphere.

[57] Chen W, Gu Z, Wen P, Li Q (2019). Degradation of refractory organic contaminants in membrane concentrates from landfill leachate by a combined coagulation–ozonation process. Chemosphere.

[58] Rivas F (2003). Stabilized leachates: Ozone-activated carbon treatment and kinetics. Water Res..

[59] Gottschalk C, Libra JA, Saupe A (2009). Ozonation of Water and Waste Water.

[60] Silva AC, Dezotti M, Sant’Anna GL (2004). Treatment and detoxification of a sanitary landfill leachate. Chemosphere.

[61] Nayeri D, Mousavi SA (2022). A comprehensive review on the coagulant recovery and reuse from drinking water treatment sludge. J. Environ Manag..

[62] Li W, Hua T, Zhou Q, Zhang S, Li F (2010). Treatment of stabilized landfill leachate by the combined process of coagulation/flocculation and powder activated carbon adsorption. Desalination.

[63] Amor C (2015). Mature landfill leachate treatment by coagulation/flocculation combined with Fenton and solar photo-Fenton processes. J. Hazard Mater..

[64] Bellouk H (2022). Performance of coagulation–flocculation followed by ultra-violet/ultrasound activated persulfate/hydrogen peroxide for landfill leachate treatment. Sci. Afr..

